# Classification Performance of General-Purpose Multimodal AI Chatbots Versus Expert Radiologists in the Winter Classification of Impacted Mandibular Third Molars on CBCT-Derived Images

**DOI:** 10.3390/diagnostics16182904

**Published:** 2026-09-09

**Authors:** Ahmet Faruk Erturk, Merve Yelken Kendirci, Oyun Erdene Batgerel, Ilknur Ozcan

**Affiliations:** 1 Department of Oral and Maxillofacial Radiology, Faculty of Dentistry, Biruni University, Istanbul 34010, Turkey; 2 Department of Restorative Dentistry, Faculty of Dentistry, Biruni University, Istanbul 34010, Turkey

**Keywords:** artificial intelligence, chatbot, classification performance, cone-beam computed tomography, impacted mandibular third molar, winter classification, oral and maxillofacial radiology

## Abstract

**Background/Objectives:** Cone-beam computed tomography (CBCT) provides three-dimensional anatomical detail that is used when classifying impacted mandibular third molars, yet the classification performance of general-purpose multimodal artificial intelligence (AI) chatbot services in this task remains unexplored. This study compared the Winter classification performance of five general-purpose multimodal AI chatbot services with that of expert oral and maxillofacial radiologists using identical standardized CBCT-derived image composites. **Methods:** A total of 114 impacted mandibular third molars were retrospectively evaluated. For each tooth, a standardized composite of axial, coronal and sagittal multiplanar reconstructions together with one three-dimensional surface rendering was exported as a single JPEG image and submitted to five multimodal AI chatbot services (ChatGPT, Gemini, Copilot, Perplexity and Grok) through their web interfaces using an identical structured prompt that named the target tooth by its FDI number (38 or 48). Each impacted tooth was submitted as a separate case in a separate cleared session; a composite was never used to query two teeth simultaneously. Two board-certified oral and maxillofacial radiologists independently classified the same JPEG composites to establish an adjudicated reference standard. Accuracy, balanced accuracy, macro-averaged recall, precision and F1 score, category-level sensitivity and specificity with exact 95% confidence intervals (CIs) and Cohen’s kappa were calculated, and the accuracy of each service was compared with the majority-class (no-information) baseline. **Results:** The adjudicated reference standard comprised mesioangular (*n* = 28), distoangular (*n* = 9), vertical (*n* = 25), horizontal (*n* = 46) and inverted (*n* = 6) impactions; no transverse case occurred, so the six-category spectrum was incomplete. Inter-observer agreement between the two radiologists was 80.7% (92/114) with Cohen’s kappa 0.688 (95% CI 0.571–0.805). Accuracy was 41.2% (95% CI 32.6–50.4) for ChatGPT, 30.7% (23.0–39.7) for Copilot and 24.6% (17.6–33.2) for Gemini, Perplexity and Grok. No service exceeded the 40.4% majority-class baseline (ChatGPT versus baseline, *p* = 0.85). Balanced accuracy was 23.8% or lower for every service, and agreement with the reference standard was slight to none (kappa −0.003 to 0.097; all 95% CIs included zero). No service produced a correct distoangular (0/9; 95% CI 0.0–33.6) or inverted (0/6; 95% CI 0.0–45.9) classification, although these categories were represented by very few cases and the corresponding estimates are therefore highly imprecise. **Conclusions:** Within the specific web interfaces, prompt, image composites and testing period evaluated, general-purpose multimodal AI chatbot services did not classify impacted mandibular third molars according to the Winter system more accurately than a trivial majority-class classifier, and their agreement with an expert-adjudicated reference standard was negligible. These findings apply to the tested consumer chatbot services and to static CBCT-derived composites and should not be generalized to all multimodal AI models or to task-specific dental AI systems.

## 1. Introduction

Mandibular third molars are the last teeth to erupt in the dental arch and frequently remain impacted due to insufficient space in the retromolar region or abnormal eruption pathways. Consequently, third molars exhibit a higher rate of impaction compared to other teeth. It has been reported that the prevalence of impacted third molars is approximately 36–37% at the individual level and roughly 46% at the tooth level. Evidence suggests that these teeth are more frequently impacted in the mandible, often leading to clinically significant complications [1,2]. Various classification systems have been developed to systematically evaluate the position of impacted third molars. Among these, the Winter classification and the Pell and Gregory classification are the most widely utilized. While the Winter classification defines the angulation of the tooth, the Pell and Gregory system assesses the relationship of the tooth to the mandibular ramus and its depth relative to the occlusal plane. These classifications provide essential clinical data for predicting surgical difficulty and informing treatment planning [1]. Throughout the present study, the term Winter classification refers exclusively to the angulation, or orientation, of the impacted tooth relative to the long axis of the adjacent erupted second molar. Depth of impaction and the relationship of the tooth to the mandibular ramus or to the mandibular canal are distinct anatomical assessments that belong to the Pell and Gregory system and to canal-proximity grading, and they were not evaluated here.

While traditional panoramic radiography is conventionally used for the assessment of impacted third molars, two-dimensional imaging modalities are limited by factors such as distortion and the superposition of anatomical structures. In contrast, cone-beam computed tomography (CBCT) enables a more detailed three-dimensional evaluation of anatomical structures, including the teeth, alveolar bone, and the mandibular canal. CBCT imaging can provide higher accuracy and sensitivity compared to panoramic radiography, particularly in assessing the relationship between the mandibular third molar and the mandibular canal [3]. In recent years, the application of artificial intelligence, specifically deep learning-based methods, has increased rapidly in medical image analysis. Algorithms such as Convolutional Neural Networks (CNN) have demonstrated successful results with high accuracy rates in tasks such as the automated detection and classification of teeth, as well as the segmentation of anatomical structures in dental radiographs. By providing diagnostic support to clinicians, these systems reduce analysis time and contribute to the minimization of human-based observational errors [4]. The high data volume and three-dimensional nature of CBCT images, in particular, can make manual evaluation time-consuming. Consequently, AI-based automated analysis methods offer significant potential for tooth segmentation, the determination of anatomical relationships, and the automated execution of radiological classifications. The integration of AI algorithms in this field could facilitate faster and more objective assessments by contributing to the development of clinical decision-support systems [5,6].

Artificial intelligence applications in the field of dentistry are relatively new. The majority of existing studies are limited to tasks such as tooth detection, caries diagnosis, or the segmentation of anatomical structures using panoramic radiographs. AI research based on CBCT images remains limited, with most studies reporting only the performance of a specific algorithm without incorporating clinically meaningful classification systems. In particular, there is a paucity of literature comparatively evaluating the angulation-based classification of impacted mandibular third molars using different AI systems. This gap is notable given that anatomical parameters such as ramus height and gonial angle have been shown to influence Pell & Gregory/Winter impaction patterns [7], underscoring that Winter classification is not a purely visual task but one that depends on subtle angular relationships. A broader trend toward AI-assisted decision support in the management of impacted third molars has also been described [8]; these adjacent tasks, however, differ fundamentally from the categorical image interpretation examined here and are therefore only referred to for context. Accordingly, the following hypotheses were prespecified, each linked to an explicitly identified analysis. H0: The classification accuracy of each AI chatbot service does not differ from the majority-class (no-information) baseline of the study sample, and the five services do not differ from one another; these components were tested by an exact binomial test against the baseline proportion and by Cochran’s Q test on case-level correct/incorrect outcomes, respectively. H1: The classification accuracy of the AI chatbot services is lower than the level of agreement achieved between the two expert radiologists; because inter-reader agreement is not an accuracy estimate, this comparison was assessed descriptively with 95% confidence intervals rather than by a formal significance test. H2: Classification accuracy differs among the AI chatbot services; this was tested by Cochran’s Q test followed by pairwise McNemar tests with Holm adjustment for multiplicity.

## 2. Materials and Methods

**Figure 1:**
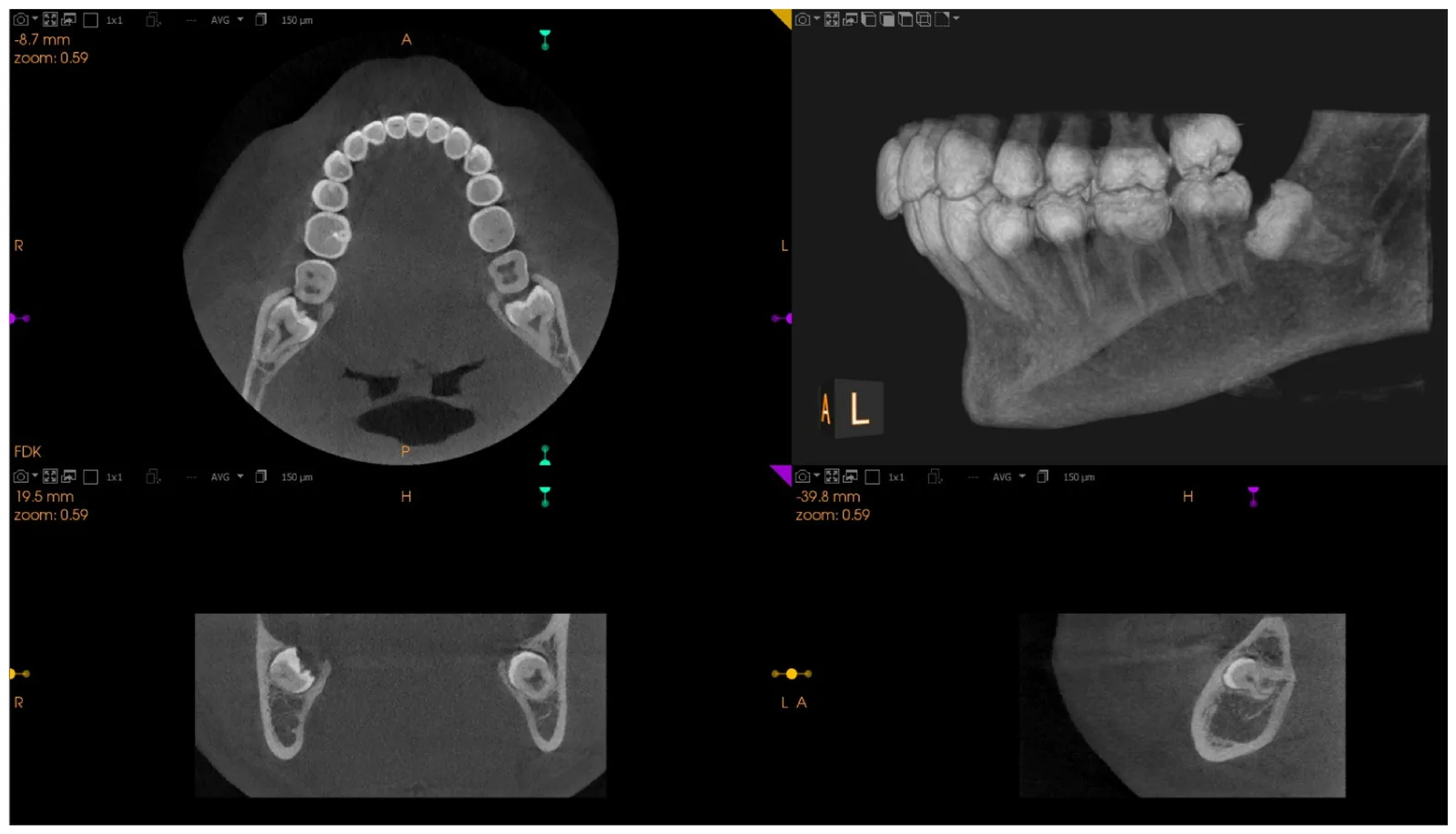
Representative example of a composite image exactly as submitted to the AI chatbot services and to the expert readers, showing the fixed two-by-two panel layout: axial (**upper left**), coronal (**upper right**) and sagittal (**lower left**) multiplanar reconstructions with a three-dimensional surface rendering (**lower right**). No marker, annotation or arrow was applied to any panel; the target tooth was specified verbally in the prompt by its FDI number.

This study was conducted in accordance with the principles of the Declaration of Helsinki (2008) and was approved by the Biruni University Scientific Research Ethics Committee on 26 January 2026 (approval number: BIAEK/17-33). Written informed consent for the research use of anonymized imaging data was obtained from all subjects whose examinations were included, and the ethics approval explicitly covered the submission of fully deidentified, non-identifiable image composites to third-party consumer AI chatbot services. The study is reported in accordance with the STARD 2015 recommendations for diagnostic accuracy studies [9] and with the CLAIM checklist for artificial intelligence in medical imaging [10]. The study population consisted of patients aged 18 years and older who were referred to the Department of Oral and Maxillofacial Radiology at Biruni University Faculty of Dentistry and who had undergone CBCT imaging for various dental indications. Examinations performed during the study period were screened retrospectively and consecutively against the eligibility criteria defined below until the prespecified sample size had been reached. A total of 114 impacted mandibular third molars from 114 patients were included in the final analysis. Each impacted third molar was treated as an independent case. When a patient presented with bilateral impacted mandibular third molars, one tooth was selected at random for inclusion, and the right and the left tooth were in every instance prepared, submitted and scored separately, each in its own cleared session; a single image composite was never used to ask about two teeth at the same time. Cases were eligible for inclusion if they featured impacted right or left mandibular third molars (teeth 38 or 48) with complete root development, a fully erupted mandibular second molar serving as the reference landmark for Winter classification, and complete visibility of the impacted tooth, the anterior border of the ramus and the second molar within the tomographic field of view. CBCT images were required to meet minimum diagnostic quality standards, defined as sufficient spatial resolution, contrast and brightness to allow unambiguous identification of the anatomical landmarks. Cases were excluded if they exhibited pathological conditions associated with the impacted tooth that could distort the anatomical position or the surrounding bone levels (cysts, tumors or follicular enlargement greater than 3 mm); image quality degradation attributable to patient motion artifacts or to metal scatter artifacts from restorations or prostheses; absence or impaction of the mandibular second molar; imaging artifacts from orthodontic brackets or wires; a history of trauma, surgery or bone resection in the mandibular region; incomplete root development (open apex); or a syndromic diagnosis. Raw CBCT data were acquired in DICOM format using a standardized imaging protocol and were fully anonymized prior to analysis by removing all patient-identifying metadata, in compliance with the Declaration of Helsinki and with Turkish Personal Data Protection Law No. 6698 (KVKK). Deidentification removed all direct and indirect identifiers from the DICOM headers and from the exported images; the exported composites contained no burned-in patient information, and no facial or full-skull rendering that could permit re-identification was produced. The data-governance arrangements applying to the chatbot interfaces are described in the following section. For each case, three multiplanar reconstruction views, axial, coronal and sagittal, were systematically selected to demonstrate the long axis of the impacted tooth in relation to the adjacent second molar, which constitutes the anatomical basis of the Winter classification. In addition, a three-dimensional surface reconstruction image was generated to provide spatial orientation. All slice selections were performed by a single oral and maxillofacial radiologist who was not one of the two reference readers and who was blinded to the reference classification at the time of image preparation. The axial view was set at the level of the cementoenamel junction of the second molar, the sagittal view was oriented along the mesiodistal long axis of the impacted tooth, and the coronal view was oriented perpendicular to that axis. The four views were exported from the CBCT software (Carestream CS9600 Dental LLC, Atlanta, GA, USA) at the highest resolution and the lowest compression level offered by its export dialogue, so that no visible compression artifact was introduced, and were assembled without resampling into a single composite image using a fixed two-by-two panel layout: the axial view in the upper left, the coronal view in the upper right, the sagittal view in the lower left and the three-dimensional surface rendering in the lower right position. Panel arrangement, field of view, cropping boundaries, magnification, window width and window level, and the rendering parameters of the three-dimensional reconstruction (segmentation threshold, camera angle and illumination) were held constant for every case. Left and right orientation labels were retained on the axial and three-dimensional panels; no other annotation, marker, arrow or post-processing filter was applied to any panel. Figure 1 is an unmodified example of a composite exactly as submitted to the chatbot services and to the expert readers.

The selection of multimodal AI chatbot services was based on publicly reported global usage share (Statcounter (2025) global usage data [11]). Five general-purpose multimodal AI chatbot services were included: ChatGPT (OpenAI, San Francisco, CA, USA), Gemini (Google, Mountain View, CA, USA), Copilot (Microsoft, Redmond, WA, USA), Perplexity (Perplexity AI, San Francisco, CA, USA) and Grok (xAI, Palo Alto, CA, USA). It must be emphasized that these are consumer chatbot services rather than reproducibly identified, version-pinned models. Web interfaces may apply internal model routing and may change their backend without public notice, and several of them do not expose a fixed underlying model to the user. Consequently, conducting all submissions within a single, narrow data-collection window reduces but does not eliminate confounding from model updates or routing changes, and the results must be read as the behavior of these interfaces during the stated testing window rather than as the behavior of any named model. To make the evaluation as reproducible as the interfaces allow, the model or version identifier displayed in each interface, the model-selection setting, the session type, the state of the image-analysis capability and the completeness of the returned responses are reported in Table 1. All services were accessed exclusively through their respective web interfaces (chatgpt.com, gemini.google.com, copilot.microsoft.com, perplexity.ai and grok.com) without API integration and using the free or standard subscription tier of each service, and all five were tested within the same data-collection window, January to February 2026, so that the interfaces were exercised under comparable conditions. Image upload and image analysis were explicitly enabled and verified for every submission, and every case was entered in a new chat with no prior context. For each case, the identical composite image was submitted to all five services together with the following structured prompt, in which the target tooth was named explicitly by its FDI number: “You are an expert dentist. Examine the attached radiographic image and classify tooth [38 or 48, as applicable] according to the Winter classification (Mesioangular, Distoangular, Vertical, Horizontal, Inverted, Transverse). State only the classification result.” The target tooth was therefore identified verbally rather than by a graphical marker, and no annotation, arrow or highlight was placed on the images. The six category names were presented in the same fixed order in every prompt; sensitivity of the responses to the order in which the categories are listed was not evaluated and is acknowledged as a limitation of prompt robustness. To prevent contextual carry-over between cases, the full conversation history of each model was cleared before each new case was submitted. This session-clearing protocol was applied uniformly across all models and all cases. Where a service returned an ambiguous, incomplete or non-classifiable first response, for example a narrative explanation instead of a single category label, the identical prompt was resubmitted once within the same cleared session; every case ultimately yielded a classifiable category label from every service, so no response had to be scored as non-classifiable. Because a second attempt was permitted only after a non-compliant first response, this protocol carries a risk of differential retry bias, which is acknowledged in the Limitations Section.

Every case was submitted in a newly opened chat with no prior context, and no temporary or private session mode was used. Because these are consumer chatbot services, the identifiers displayed by the interfaces do not guarantee a fixed underlying model, and internal routing may vary between submissions.

The radiological reference standard was established by two oral and maxillofacial radiology specialists, each with a minimum of three years of post-specialization clinical and academic experience in CBCT-based dental imaging. Critically, both specialists classified exactly the same static JPEG composite images that were submitted to the AI chatbot services; no interactive multiplanar navigation of the original DICOM volumes was performed for the purpose of establishing the reference standard, and the target tooth was identified to the readers by the same FDI number used in the chatbot prompt. Human and machine readers therefore received identical diagnostic information in an identical format, so that the comparison is not confounded by differences in the amount or the modality of the information available. Both specialists evaluated all cases independently under blinded conditions, with no access to each other’s classifications or to the AI-generated outputs. Cases were presented in a randomized order. Classification followed prespecified operational definitions. The angle was measured in the sagittal plane between the long axis of the impacted third molar and the long axis of the adjacent erupted mandibular second molar, the long axis being defined as the line connecting the midpoint of the occlusal surface to the midpoint of the furcation or, in single-rooted teeth, to the root apex. The angular thresholds were as follows: mesioangular, crown tilted mesially with an inter-axial angle of 11 to 70 degrees; distoangular, crown tilted distally with an inter-axial angle of 11 to 70 degrees; vertical, inter-axial angle of 10 degrees or less; horizontal, inter-axial angle of 71 to 110 degrees; inverted, inter-axial angle greater than 110 degrees with the crown directed apically; and transverse, long axis of the impacted tooth oriented buccolingually on the axial and coronal views, that is, approximately perpendicular to the sagittal plane of the arch. The buccoangular and linguoangular orientations that appear in some formulations of the Winter system were subsumed under the transverse category, because both denote a predominantly buccolingual long-axis orientation and both are exceedingly rare. This six-category taxonomy was fixed a priori and was presented identically to the human readers and to the chatbot services. Depth of impaction and the relationship of the tooth to the ramus or to the mandibular canal were not assessed. Evaluations were performed on a calibrated diagnostic-grade monitor under standardized ambient lighting conditions. To assess intra-observer reliability, each specialist re-evaluated a randomly selected 20% subset of cases (*n* = 23) at an interval of at least two weeks after the initial assessment. In cases of inter-observer disagreement, the final classification was determined by the more senior specialist, defined as the evaluator with greater clinical seniority and higher academic rank. This conflict-resolution protocol was established a priori, prior to data collection, to eliminate the risk of post hoc adjudication bias. Because this procedure resolves disagreements in favor of one of the two contributing readers rather than through an independent third assessment, the resulting labels are referred to throughout as an adjudicated reference standard and not as a gold standard.

The minimum required sample size was calculated a priori using G*Power 3.1 software (Heinrich Heine University, Dusseldorf, Germany). The primary outcome was the case-level agreement between a chatbot service and the adjudicated reference standard across the six Winter categories. The calculation used the chi-square family test with a medium effect size of w = 0.36, df = 5, a two-sided alpha of 0.05 and a target power of 0.80, which yielded a required sample of 99 cases; this was rounded to a minimum of 100 impacted third molars, and 114 were ultimately analyzed. No assumption of a uniform category distribution was made; the naturally occurring distribution of the clinical population was accepted, and the consequences of the resulting class imbalance are addressed in the analysis plan below and in the Limitations. All statistical analyses were performed using IBM SPSS Statistics version 26.0 (IBM Corp., Armonk, NY, USA) and R version 4.4.1, and all estimates were recalculated directly from the case-level dataset. Continuous demographic variables were described using means with standard deviations together with the observed range, their distributional properties having been assessed with the Shapiro–Wilk test. The classification performance of each AI chatbot service was characterized by calculating overall accuracy, balanced accuracy (the unweighted mean of the category-level recalls over the categories represented in the sample), macro-averaged precision and macro-averaged F1 score, and category-level sensitivity and category-level specificity, with all estimates reported alongside 95% confidence intervals. Confidence intervals for overall accuracy were calculated by the Wilson score method and those for category-level sensitivity and specificity by the exact Clopper–Pearson method, the latter being appropriate for the very small denominators of the rare categories. Because precision, recall and F1 score are not interpretable as single numbers for a nominal multiclass outcome unless the averaging scheme is stated, all such values are reported as unweighted macro-averages over the five categories represented in the reference standard; the transverse category, for which no case occurred, is excluded from every average and its sensitivity is not estimable. Positive and negative likelihood ratios, negative predictive value and the discordant odds ratio were not computed, because a single such value has no defined interpretation for a six-category nominal outcome unless an explicit one-versus-rest formulation is specified. Because the reference categories are markedly imbalanced, overall accuracy alone can be misleading; the accuracy of each service was therefore also compared with the majority-class, or no-information, baseline, defined as the proportion of the most frequent reference category, using an exact binomial test. Differences in case-level correct/incorrect outcomes among the five services, which are paired because every service classified the same cases, were tested with Cochran’s Q test, followed by pairwise exact McNemar tests with Holm correction for multiplicity. A standard McNemar test does not assess agreement between two six-category classifications and was therefore not used for that purpose; where the marginal distributions of the predicted and the reference categories were compared, the Stuart–Maxwell test of marginal homogeneity and Bowker’s test of symmetry were applied. Agreement between each AI chatbot service and the adjudicated reference standard, and between the two expert readers, was quantified using Cohen’s kappa coefficient with asymptotic 95% confidence intervals. The kappa values were interpreted according to the Landis and Koch scale: ≤0.20 slight, 0.21–0.40 fair, 0.41–0.60 moderate, 0.61–0.80 substantial, and >0.80 almost perfect agreement; in addition, kappa values at or below zero were classified as indicating poor (no better than chance) agreement, consistent with common convention in the diagnostic-accuracy literature. Accuracy for right (tooth 48) and left (tooth 38) third molars was compared with Fisher’s exact test, these two groups being independent. The level of statistical significance was set at *p* < 0.05 for all analyses.

## 3. Results

Analysis of the demographic data shows that 114 patients, with a mean age of 25.9 ± 6.9 years (range 18 to 53 years), were included in the study. Female patients constituted 69.3% of the sample and male patients 30.7%. The Shapiro–Wilk test applied to the age variable indicated that the data did not follow a normal distribution, and age is therefore additionally summarized by its observed range (Table 2). In the analyses conducted to determine the reliability of the radiological evaluations, the two oral and maxillofacial radiology specialists agreed on the Winter classification in 92 of the 114 cases (80.7%), with a Cohen’s kappa coefficient of 0.688 (95% CI 0.571–0.805), which corresponds to substantial agreement. The decisions of the radiologist with greater clinical experience and academic seniority were adopted as the adjudicated reference labels for the 22 cases in which the two readers disagreed, following the rule fixed before data collection. To test intra-observer consistency over time, a test–retest analysis was performed on a randomly selected 20% subset of the dataset (*n* = 23), yielding intra-observer kappa coefficients of 0.854 (95% CI 0.680–1.000) for the first radiologist and 0.812 (95% CI 0.618–1.000) for the second. Intra-observer agreement was therefore substantial to almost perfect, although the confidence intervals are wide because only 23 cases were reassessed. An inter-observer kappa of 0.688 indicates a substantial but not near-perfect level of concordance, and the reference standard is accordingly regarded as adequately reliable for the present comparison (Table 2).

The distribution of Winter classification categories according to the adjudicated reference standard is presented in Table 3. Mesioangular (*n* = 28), distoangular (*n* = 9), vertical (*n* = 25), horizontal (*n* = 46) and inverted (*n* = 6) impactions were represented, whereas no transverse case occurred in the sample; the study therefore evaluates performance within a naturally occurring but incomplete clinical spectrum, and sensitivity for the transverse category could not be estimated at all. Among the multimodal chatbot services included in the study, ChatGPT achieved the highest overall accuracy. It classified 47 of 114 cases in accordance with the reference standard, an accuracy of 41.2% (95% Wilson CI 32.6–50.4), with Cohen’s kappa 0.097 (95% CI −0.042 to 0.235), the confidence interval of which includes zero; agreement with the reference standard was therefore slight at best and not statistically distinguishable from chance. Its accuracy did not differ from the 40.4% majority-class baseline of the sample (exact binomial *p* = 0.85), and its balanced accuracy was 23.5%. When analyzed by category, ChatGPT demonstrated a more balanced distribution of predictions compared to other services, particularly in the detection of impacted teeth in the horizontal position, with a sensitivity of 78.3% (95% CI 63.6–89.1) and a specificity of 42.6% (95% CI 30.7–55.2). However, it produced no correct classification in the distoangular (0 of 9; 95% CI 0.0–33.6) or inverted (0 of 6; 95% CI 0.0–45.9) categories, and the transverse category could not be assessed because no such case was present. These three categories were represented by very few cases or by none at all, so the observed failure cannot be attributed to the intrinsic difficulty of these orientations; the corresponding estimates are extremely imprecise, as the upper confidence limits of 33.6% and 45.9% show, and are compatible with a wide range of true sensitivities (Table 4 and Table 5).

The Microsoft Copilot service exhibited the second-highest accuracy following ChatGPT, classifying 35 of 114 cases in accordance with the reference standard, an accuracy of 30.7% (95% CI 23.0–39.7), with a balanced accuracy of 23.8%. Cohen’s kappa was 0.077 (95% CI −0.036 to 0.190), again including zero, and the accuracy was significantly lower than the 40.4% majority-class baseline (exact binomial *p* = 0.036). Copilot assigned the mesioangular label to 105 of the 114 cases, so that its perfect mesioangular sensitivity of 100% (95% CI 87.7–100.0) was obtained at the cost of a mesioangular specificity of only 10.5% (95% CI 4.9–18.9). Sensitivity was 8.0% (95% CI 1.0–26.0) for vertical and 10.9% (95% CI 3.6–23.6) for horizontal impactions, and no distoangular (0 of 9) or inverted (0 of 6) case was correctly classified, with the imprecision noted above.

The Google Gemini service produced classifications that were statistically independent of the reference standard, with an accuracy of 24.6% (28 of 114; 95% CI 17.6–33.2) and a kappa of 0.002 (95% CI −0.102 to 0.107). Accuracy was significantly below the 40.4% majority-class baseline (exact binomial *p* < 0.001), and balanced accuracy was 20.0%. Examination of the service’s outputs shows a sensitivity of 100% (95% CI 87.7–100.0) for mesioangular classification that is entirely attributable to the service labeling nearly all images as “mesioangular” (109 of 114 responses). This tendency resulted in a very low specificity value of 5.8% (95% CI 1.9–13.0) and led to a high rate of false-positive results. Gemini’s outputs therefore showed no agreement with the reference standard beyond chance and are consistent with a fixed response bias rather than with image interpretation.

The Grok service showed no agreement with the reference standard beyond chance, with an accuracy of 24.6% (28 of 114; 95% CI 17.6–33.2) and a kappa of 0.001 (95% CI −0.104 to 0.106); its accuracy was significantly below the 40.4% majority-class baseline (exact binomial *p* < 0.001) and its balanced accuracy was 20.0%. Its ability to distinguish between the Winter categories was correspondingly limited. Grok assigned the mesioangular label to 111 of the 114 cases and the vertical label to the remaining three, so that its correct classifications comprised the mesioangular cases only; its case-level correct/incorrect pattern was identical to that of Gemini (McNemar *p* = 1.000).

The Perplexity service achieved an overall accuracy of 24.6% (28 of 114; 95% CI 17.6–33.2) in the Winter classification task; however, its agreement with the reference standard followed a negative trend, with a kappa of −0.003 (95% CI −0.108 to 0.102). This negative value indicates that the agreement between the service’s predictions and the adjudicated reference labels was marginally lower than chance expectation, although the confidence interval spans zero and the point estimate should not be over-interpreted; accuracy was significantly below the 40.4% majority-class baseline (exact binomial *p* < 0.001) and balanced accuracy was 19.4%. Although Perplexity generated correct predictions for 26 of 28 mesioangular cases (92.9%; 95% CI 76.5–99.1) and for 2 of 46 horizontal cases (4.3%; 95% CI 0.5–14.8), it assigned the mesioangular label to 106 of the 114 cases, so that its mesioangular specificity was only 7.0% (95% CI 2.6–14.6); this pattern is again more consistent with a dominant response bias than with image interpretation.

The overall classification performance of the five chatbot services against the adjudicated reference standard is summarized in Table 6. ChatGPT reached the highest accuracy, 41.2% (95% CI 32.6–50.4), followed by Copilot at 30.7% (95% CI 23.0–39.7), while Gemini, Grok and Perplexity each reached 24.6% (95% CI 17.6–33.2). The single most important interpretive finding is that these values must be read against the no-information rate of the sample. Horizontal impactions accounted for 46 of 114 cases, so a trivial classifier that assigned every case to the majority class would achieve an accuracy of 40.4% (95% CI 31.8–49.5). ChatGPT’s accuracy did not differ from this baseline (exact binomial *p* = 0.85), and the accuracies of Copilot (*p* = 0.036), Gemini, Grok and Perplexity (each *p* < 0.001) were significantly below it. None of the evaluated services therefore performed better than a classifier that ignores the image entirely, and the apparent ranking of ChatGPT as the leading service does not represent evidence of image interpretation. This reading is reinforced by the balanced accuracies, which were 23.5% for ChatGPT, 23.8% for Copilot, 20.0% for Gemini and Grok and 19.4% for Perplexity, and by the macro-averaged F1 scores, which ranged from 8.1% to 18.5% (Table 6). It is further supported by the marked response concentration of four of the five services, which assigned a single label to between 92% and 97% of all cases. Cochran’s Q test indicated that case-level correct/incorrect outcomes were not homogeneous across the five services (Q = 22.71, df = 4, *p* < 0.001), so the null hypothesis of equal performance among services (H0, second component) was rejected; however, after Holm adjustment for the ten pairwise comparisons, no individual pairwise McNemar test remained significant (all adjusted *p* ≥ 0.127), and the unadjusted comparisons that favored ChatGPT and Copilot over the three lowest-performing services (*p* = 0.013 to 0.016) should therefore be regarded as exploratory (Table 7). Because no service exceeded the no-information rate, the first component of H0, equality with the majority-class baseline, was not rejected for ChatGPT, and H2 receives only partial and imprecise support. Regarding H1, all model accuracies fell below the 80.7% agreement observed between the two expert readers, but this contrast is descriptive only: inter-reader agreement is a measure of concordance between two observers and is not an estimate of expert diagnostic accuracy, and it cannot be compared directly with model accuracy measured against a reference standard that was itself derived in part from those same two readers. ChatGPT’s horizontal sensitivity of 78.3% (95% CI 63.6–89.1) is compatible with a preference for the majority category rather than with a specific capacity to recognize horizontal geometry, since the same service also produced a horizontal specificity of only 42.6%. Sensitivity for distoangular and inverted impactions was 0% for every service, but with only nine and six cases respectively the upper 95% confidence limits reach 33.6% and 45.9%, and no transverse case was available at all; these findings therefore describe the behavior of the services within this small and incomplete subset and do not establish that the services are categorically unable to recognize these orientations. Accuracy did not differ significantly between right (tooth 48) and left (tooth 38) third molars for any service (Fisher’s exact test, all *p* ≥ 0.545; Table 8).

The marginal distributions of the predicted and the reference categories were additionally compared for each service using the Stuart–Maxwell test of marginal homogeneity. The test was significant for every service (all *p* < 0.001), confirming that the distribution of labels produced by the services differed systematically from the distribution of the reference categories rather than differing only in the allocation of individual cases. The magnitude of this discrepancy is evident in the lower block of Table 4: against a reference distribution containing 46 horizontal and 28 mesioangular impactions, Gemini, Grok, Perplexity and Copilot assigned the mesioangular label to between 105 and 111 of the 114 cases.

## 4. Discussion

The primary finding of this study is that general-purpose multimodal artificial intelligence chatbot services did not, in this sample, classify impacted mandibular third molars according to the Winter system more accurately than a classifier that assigns every case to the most frequent category. Although ChatGPT achieved the numerically highest accuracy among the services tested, its 41.2% did not differ from the 40.4% no-information rate of the dataset, and the agreement of every service with the adjudicated reference standard was slight or absent, with all kappa confidence intervals including zero. Taken together with the strong concentration of the outputs on one or two labels, these results are more consistent with a response-category bias than with genuine interpretation of the submitted images. The findings are specific to the web interfaces, versions, prompt, image composites and testing window used here, and they do not license conclusions about all multimodal AI systems; in particular, they say nothing about task-specific models trained on annotated dental imaging. Whether these services could become useful in this task through domain-specific adaptation is an open question that the present design cannot address.

It is observed that the performance of artificial intelligence in dental radiology varies significantly depending on the task type and model architecture. Kılıç et al. reported high sensitivity (0.9804), precision (0.9571), and F1 scores (0.9686) using a task-specific Faster R-CNN-based model developed for the automated detection and numbering of primary teeth in pediatric panoramic radiographs. In contrast, the general-purpose multimodal chatbot services evaluated in our study showed no agreement beyond chance with the adjudicated reference standard in CBCT-based Winter classification. This discrepancy suggests that while deep learning algorithms specifically trained for a particular task can exhibit superior performance in relatively more defined and standardized radiological tasks, general-purpose multimodal models have not yet achieved the same level of success in dentomaxillofacial tasks that require more complex anatomical interpretation and classification [4].

The systematic review by Assiri et al. demonstrated that artificial intelligence applications for impacted mandibular third molars primarily focus on inferior alveolar nerve canal relationships, impaction positions, and treatment planning, with existing evidence generally indicating a moderate-to-good level of performance. However, the same review emphasizes that AI applications are still in the early stages of development and that further studies are required. The low accuracy and agreement values obtained in our study are consistent with that assessment, although the reviewed applications are task-specific systems and are therefore not directly comparable with the consumer chatbot services evaluated here. A CBCT-based task requiring angular and anatomical interpretation, such as the Winter classification, appears to pose a greater challenge for general-purpose multimodal services than the task-specific neural network applications frequently reported as successful in the literature [12].

In the task-specific CBCT-based deep learning system developed by Orhan et al., the detection rate for impacted third molars was reported as 86.2%, tooth numbering accuracy as 96.9%, the correct determination of root number as 78.6%, and the correct identification of the number of canals as 68.1%; furthermore, the kappa values were 0.762 for the relationship between the mandibular third molar and the inferior alveolar canal and 0.620 for the number of roots. In contrast, in our study, the overall accuracy of ChatGPT—the highest-performing model regarding Winter classification—was 41.2% (95% CI 32.6–50.4), with a Cohen’s kappa of 0.097 (95% CI −0.042 to 0.235). Copilot reached an accuracy of 30.7% and a kappa of 0.077, while the Gemini, Grok, and Perplexity models each remained at an accuracy level of 24.6%, none of which exceeded the 40.4% majority-class baseline of the sample. Moreover, while the agreement rate between the two expert radiologists was 80.7% with a kappa of 0.688, the chatbot services did not approach this level, although inter-reader agreement is a measure of concordance rather than of accuracy and the two quantities are not directly comparable. When evaluated numerically, it is evident that task-specific CNN-based systems demonstrate higher and clinically more meaningful performance in anatomical detection and relationship analysis on CBCT; conversely, the general-purpose chatbot services evaluated here showed no agreement beyond chance in the Winter classification task; the two studies address different tasks on different data, however, so the comparison is contextual rather than a direct performance contrast [13].

In the study by Makrygiannakis and Kaklamanos evaluating the Diagnocat™ (Diagnocat, Diagnocat Inc., San Francisco, CA, USA) software on panoramic radiographs, the sensitivity of the artificial intelligence model for detecting impacted third molars was reported as 88.89%, with a specificity of 100%, a positive predictive value of 100%, and a negative predictive value of 94.64%; the agreement between expert evaluations and AI was found to be almost perfect, with a Cohen’s kappa of 0.914. In contrast, the performance of the general-purpose multimodal services evaluated in our study for the purpose of Winter classification using CBCT-derived composites remained markedly lower. The two studies address fundamentally different tasks, however, and the comparison is offered as context rather than as evidence of a difference between systems: the task evaluated by Makrygiannakis and Kaklamanos is a binary detection problem, namely identifying the presence of an impacted third molar on a panoramic radiograph, whereas the Winter classification targeted in our study is a six-category nominal classification of the angulation of the tooth’s long axis relative to the second molar [14].

The longitudinal study by Chopra et al. is noteworthy as it demonstrates that artificial intelligence cannot answer every question regarding impacted third molars with equal success. Although the researchers evaluated 1542 mandibular third molars from 771 patients, they were unable to develop a clinically viable model to predict full eruption. In contrast, meaningful threshold values were identified for the more limited objective of uprighting; in the presence of adequate retromolar space, an initial angle of less than 32° was interpreted in favor of uprighting, and this model exhibited a 91% positive predictive value and 83% sensitivity/specificity in the training set. Furthermore, within the entire sample, the rate of full eruption was only 13.9%, and the rate of hygienic cleansability was 1.7%. Eruption prediction and categorical image classification are, however, fundamentally different tasks, and the comparison is drawn only to illustrate that AI performance in the third-molar domain is task-dependent. Similarly, in our study, the low accuracy and absent agreement shown by the general-purpose chatbot services in CBCT-based Winter classification suggest that the issue is related not only to model capability but also to the nature of the target task. In other words, the success of AI applications in the field of third molars is not uniform; sub-tasks such as longitudinal eruption prediction, angular repositioning forecasting, and existing radiological classification involve varying levels of difficulty [15].

The discrepancy between general-purpose multimodal services and task-specific systems observed in this study is likely attributable to differences in training objective rather than raw model capacity. Task-specific convolutional and transformer-based architectures are trained end-to-end on annotated CBCT or radiographic datasets for a single, well-defined output, allowing them to learn fine-grained voxel- or pixel-level cues associated with root angulation and ramus relationships. General-purpose multimodal services, by contrast, are built on models optimized for broad, largely two-dimensional natural-image understanding and conversational reasoning; when presented with static multiplanar CBCT screenshots, they lack any task-specific fine-tuning on dental radiological classification. This distinction is one possible explanation for the observed pattern of responses, in which four of the five services collapsed onto a single dominant label. It must be emphasized that the human readers in this study classified exactly the same static composites, without interactive multiplanar navigation of the DICOM volumes, and nevertheless achieved substantial inter-reader agreement. The limited performance of the chatbot services therefore cannot be attributed to the static, two-dimensional format of the input, since that constraint applied equally to both reader types. What the present design cannot distinguish is whether the services would perform better with full volumetric input, with a different prompt, or with repeated sampling, since none of these conditions were tested.

### Limitations

This study has several important limitations, which jointly constrain how far the findings can be generalized. First, and most importantly for the interpretation of the category-level results, the reference sample reflected a naturally occurring but incomplete clinical spectrum. Horizontal impactions accounted for 46 of 114 cases, whereas only nine distoangular and six inverted cases were present and no transverse case occurred at all. The complete absence of transverse cases means that sensitivity for that category could not be estimated in any way, so the six-category Winter taxonomy was not validated in full. For the distoangular and inverted categories, the observed sensitivity of 0% carries exact upper 95% confidence limits of 33.6% and 45.9% respectively, which are entirely compatible with clinically non-trivial true sensitivities. The failure of the services in these categories may therefore reflect the small or absent sample sizes as much as any intrinsic difficulty of these orientations, and the earlier characterization of them as the categories of greatest diagnostic significance is not supported by data collected here. All statements about these categories have accordingly been restricted to the small subset actually evaluated, and no claim is made that the services are categorically unable to recognize distoangular, inverted or transverse impactions.

Second, the entities evaluated are consumer chatbot services rather than reproducibly identified, version-pinned models. Their web interfaces may apply internal model routing and may alter their backend without public notice, and for some of them no fixed underlying model is exposed to the user. Restricting all submissions to a single narrow window reduces but does not eliminate this source of variation, and the interface documentation in Table 1 is the best available substitute for true version control. Any attempt to replicate this work will necessarily be a replication of the interfaces as they behave at the time of replication rather than of the specific systems evaluated here.

Third, each case was submitted once to each service. A single output does not characterize the repeatability of a stochastic system, so within-service consistency could not be quantified, and the reported accuracies should be read as single draws rather than as expected values. The protocol also permitted one resubmission, but only after a non-compliant first response, which introduces a potential differential retry bias, since a service that answered in an unusable format was effectively granted a second draw while a service that answered incorrectly but in the required format was not. In addition, only a single prompt formulation was used, in English, with the six category names always listed in the same order, so sensitivity of the outputs to prompt wording, to language and to category order remains unexamined. Repeated independent runs, randomized category order and alternative prompt formulations are the obvious priorities for any confirmatory study.

Fourth, disagreements between the two readers were resolved by the more senior reader rather than by consensus with an independent third radiologist. This procedure was fixed a priori, but it does not yield a reader-independent criterion and may transmit the systematic tendencies of one reader to the reference labels, which is why the labels are described throughout as an adjudicated reference standard rather than as a gold standard. The retest subset used for intra-observer agreement comprised only 23 cases, so the corresponding kappa estimates are imprecise. Fifth, images were submitted through publicly accessible consumer interfaces; although every composite was fully deidentified and contained no identifying information, this access route does not provide the data-processing guarantees of enterprise or API-based deployments, and the arrangements that were relied upon are described in the Institutional Review Board and Data Availability statements. Sixth, the study was conducted at a single centre with a single CBCT protocol and a single composite-image format; performance with other acquisition parameters, other panel layouts or full volumetric input was not assessed. Finally, both reader types received identical static composites, which removes the confounding of input format from the primary comparison but also means that the study does not estimate what expert radiologists would achieve with full interactive DICOM review, nor what the chatbot services would achieve with volumetric input.

## 5. Conclusions

The present study evaluated the classification performance of five general-purpose multimodal AI chatbot services in the Winter classification of impacted mandibular third molars, using standardized CBCT-derived image composites, and compared their outputs against an adjudicated expert reference standard established by trained oral and maxillofacial radiologists who classified exactly the same composites. Within this sample, none of the services classified the images more accurately than a trivial majority-class classifier: the highest accuracy, 41.2% for ChatGPT, was indistinguishable from the 40.4% no-information rate, and agreement with the reference standard was slight or absent for every service, with all kappa confidence intervals including zero. The strong concentration of outputs on one or two labels indicates that the observed accuracies are better explained by response-category bias than by interpretation of the images.

A secondary observation is that no service produced a correct distoangular or inverted classification. This observation must, however, be interpreted with caution: only nine distoangular and six inverted cases were available, no transverse case occurred, and the exact upper 95% confidence limits for these zero sensitivities reach 33.6% and 45.9%. The present data therefore describe the behavior of the services within a small and incomplete subset of the Winter spectrum and do not establish that these orientations are categorically beyond the reach of general-purpose multimodal systems, nor that they carry the greatest diagnostic significance in practice, a claim for which this study provides no evidence.

Unlike task-specific deep learning systems developed and validated for discrete radiological objectives, the services evaluated in this study were not designed for structured radiological classification. The results therefore reinforce that the integration of AI into CBCT-based diagnostic workflows must be approached with rigorous validation standards, and that performance demonstrated in general imaging tasks does not transfer predictably to specialized image-classification tasks. Because the human readers classified the identical static composites and nevertheless reached substantial inter-reader agreement, the format of the input cannot by itself account for the results obtained from the chatbot services.

In conclusion, under the specific interfaces, versions, prompt, image composites and testing window evaluated here, general-purpose multimodal AI chatbot services did not provide usable Winter classification of impacted mandibular third molars and cannot be recommended for independent use in this task. These conclusions are limited to the services and conditions tested and should not be extended to multimodal AI systems in general or to task-specific dental AI applications. Future research should focus on developing and validating task-specific AI architectures trained on large, annotated CBCT datasets with radiologist-derived ground truth, which may ultimately serve as meaningful decision-support tools within radiologist-supervised diagnostic pathways. Confirmatory evaluations of general-purpose services should include repeated independent runs, prospectively balanced representation of the rare Winter categories, prompt-robustness testing and documented version control.

## Figures and Tables

**Table 1:** Documentation of the five evaluated general-purpose AI chatbot services as presented by their web interfaces during the data-collection window.

Item	ChatGPT	Gemini	Perplexity	Copilot	Grok
Provider	OpenAI	Google	Perplexity AI	Microsoft	xAI
Web interface	chatgpt.com	gemini.google.com	perplexity.ai	copilot.microsoft.com	grok.com
Model or version identifier displayed in the interface	GPT-4o	Gemini 1.5 Pro	Perplexity Pro (interface default model)	Copilot with GPT-4	Grok-2
Model-selection setting	Model explicitly selected in the interface	Model explicitly selected in the interface	Interface default routing; no fixed underlying model exposed to the user	Interface default routing; no fixed underlying model exposed to the user	Model explicitly selected in the interface
Session type	New standard chat opened for every case; no temporary or private session	New standard chat opened for every case; no temporary or private session	New standard chat opened for every case; no temporary or private session	New standard chat opened for every case; no temporary or private session	New standard chat opened for every case; no temporary or private session
Image analysis	Explicitly enabled and verified for every submission	Explicitly enabled and verified for every submission	Explicitly enabled and verified for every submission	Explicitly enabled and verified for every submission	Explicitly enabled and verified for every submission
Response completeness	All 114 submissions returned a classifiable category label	All 114 submissions returned a classifiable category label	All 114 submissions returned a classifiable category label	All 114 submissions returned a classifiable category label	All 114 submissions returned a classifiable category label

**Table 2:** Demographic and clinical characteristics of the study population.

Variable	*n*/Mean ± SD	%
Age, years (mean ± SD)	25.9 ± 6.9	–
Age range, years	18–53	–
Sex		
Female	79	69.3
Male	35	30.7
Impacted tooth		
48 (right)	63	55.3
38 (left)	51	44.7
Total	114	100.0

SD: Standard deviation.

**Table 3:** Distribution of impacted mandibular third molars according to the Winter classification (reference standard).

Winter Classification Category	*n*	%
Mesioangular	28	24.5
Distoangular	9	7.9
Vertical	25	21.9
Horizontal	46	40.4
Inverted	6	5.3
Transverse	0	0.0
Total	114	100.0

Reference standard established by the oral and maxillofacial radiologists and adjudicated as described in the Methods. No case was classified as transverse in the study sample; the evaluated spectrum is therefore an incomplete, naturally occurring clinical spectrum rather than the full six-category taxonomy, and the majority category (horizontal) accounts for 40.4% of all cases, which defines the no-information baseline used in the analysis.

**Table 4:** Category-level sensitivity and specificity of the five AI chatbot services for Winter classification, with exact (Clopper–Pearson) 95% confidence intervals, and the distribution of the labels each service predicted.

Winter Category	ChatGPT	Gemini	Perplexity	Copilot	Grok
Sensitivity, % (95% CI)					
Mesioangular (*n* = 28)	39.3 (21.5–59.4)	100.0 (87.7–100.0)	92.9 (76.5–99.1)	100.0 (87.7–100.0)	100.0 (87.7–100.0)
Distoangular (*n* = 9)	0.0 (0.0–33.6)	0.0 (0.0–33.6)	0.0 (0.0–33.6)	0.0 (0.0–33.6)	0.0 (0.0–33.6)
Vertical (*n* = 25)	0.0 (0.0–13.7)	0.0 (0.0–13.7)	0.0 (0.0–13.7)	8.0 (1.0–26.0)	0.0 (0.0–13.7)
Horizontal (*n* = 46)	78.3 (63.6–89.1)	0.0 (0.0–7.7)	4.3 (0.5–14.8)	10.9 (3.6–23.6)	0.0 (0.0–7.7)
Inverted (*n* = 6)	0.0 (0.0–45.9)	0.0 (0.0–45.9)	0.0 (0.0–45.9)	0.0 (0.0–45.9)	0.0 (0.0–45.9)
Specificity, % (95% CI)					
Mesioangular	67.4 (56.5–77.2)	5.8 (1.9–13.0)	7.0 (2.6–14.6)	10.5 (4.9–18.9)	3.5 (0.7–9.9)
Distoangular	100.0 (96.5–100.0)	97.1 (91.9–99.4)	98.1 (93.3–99.8)	98.1 (93.3–99.8)	100.0 (96.5–100.0)
Vertical	100.0 (95.9–100.0)	100.0 (95.9–100.0)	97.8 (92.1–99.7)	100.0 (95.9–100.0)	96.6 (90.5–99.3)
Horizontal	42.6 (30.7–55.2)	97.1 (89.8–99.6)	97.1 (89.8–99.6)	100.0 (94.7–100.0)	100.0 (94.7–100.0)
Inverted	100.0 (96.6–100.0)	100.0 (96.6–100.0)	100.0 (96.6–100.0)	100.0 (96.6–100.0)	100.0 (96.6–100.0)
Transverse	100.0 (96.8–100.0)	100.0 (96.8–100.0)	100.0 (96.8–100.0)	100.0 (96.8–100.0)	100.0 (96.8–100.0)
Number of predicted labels, n (total 114)					
Mesioangular	39	109	106	105	111
Distoangular	0	3	2	2	0
Vertical	0	0	2	2	3
Horizontal	75	2	4	5	0
Inverted	0	0	0	0	0
Transverse	0	0	0	0	0

Sensitivity and specificity are percentages with exact 95% confidence intervals in parentheses. Sensitivity for the transverse category is not estimable and is therefore not listed, because no case in the reference standard belonged to that category. Denominators for sensitivity are the reference-standard counts given in Table 3 (mesioangular 28, distoangular 9, vertical 25, horizontal 46, inverted 6); denominators for specificity are 114 minus those counts. The wide intervals for the distoangular and inverted categories reflect their small sample sizes and should be taken into account in any interpretation of the zero sensitivities. The lower block gives the number of cases to which each service assigned each label; together with the sensitivities above it, it specifies the diagonal and the marginal totals of every confusion matrix.

**Table 5:** Cross-tabulation (confusion matrix) of ChatGPT predictions against the adjudicated reference standard.

Reference Standard ↓/ChatGPT Prediction →	Mesioangular	Distoangular	Vertical	Horizontal	Inverted	Transverse
Mesioangular	11	0	0	17	0	0
Distoangular	2	0	0	7	0	0
Vertical	14	0	0	11	0	0
Horizontal	10	0	0	36	0	0
Inverted	2	0	0	4	0	0
Transverse	0	0	0	0	0	0

Rows represent the adjudicated reference classification and columns the classification generated by ChatGPT. ChatGPT never predicted the distoangular, vertical, inverted or transverse categories for any case. The corresponding distributions of predicted labels for the remaining four services are given in the lower block of Table 4, which, together with the category-level sensitivities and specificities reported there, fully specifies the diagonal and the marginal totals of their confusion matrices.

**Table 6:** Classification performance of the five AI chatbot services against the adjudicated reference standard, with the majority-class baseline for comparison.

Metric	ChatGPT	Gemini	Perplexity	Copilot	Grok
Correct classifications, *n*/114	47	28	28	35	28
Accuracy, % (95% CI)	41.2 (32.6–50.4)	24.6 (17.6–33.2)	24.6 (17.6–33.2)	30.7 (23.0–39.7)	24.6 (17.6–33.2)
Balanced accuracy (macro-averaged recall), %	23.5	20.0	19.4	23.8	20.0
Macro-averaged precision, %	15.2	5.1	14.9	45.3	5.0
Macro-averaged F1 score, %	18.5	8.2	9.4	15.3	8.1
Cohen’s kappa (95% CI)	0.097 (−0.042 to 0.235)	0.002 (−0.102 to 0.107)	−0.003 (−0.108 to 0.102)	0.077 (−0.036 to 0.190)	0.001 (−0.104 to 0.106)
*p* (kappa = 0)	0.173	0.968	0.957	0.180	0.986
Strength of agreement	Slight	None	None	Slight	None
*p* versus majority-class baseline (40.4%) *	0.85	<0.001	<0.001	0.036	<0.001

* Exact binomial test of the model accuracy against the no-information rate of the sample, which is the proportion of the most frequent reference category (horizontal, 46/114 = 40.4%; 95% CI 31.8–49.5). CI = confidence interval; accuracy intervals are Wilson score intervals. Macro-averaged values are unweighted means over the five categories represented in the reference standard; the transverse category is excluded because no case occurred. Positive and negative likelihood ratios, negative predictive value and the discordant odds ratio are not reported because they have no defined interpretation as single values for a six-category nominal outcome.

**Table 7:** Comparison of case-level correct/incorrect outcomes among the five AI chatbot services.

Comparison	Discordant Pairs (b/c)	*p*	Holm-Adjusted *p*
Cochran’s Q test across all five services	-	Q = 22.71, df = 4, *p* < 0.001	-
ChatGPT vs. Gemini	36/17	0.013	0.127
ChatGPT vs. Grok	36/17	0.013	0.127
ChatGPT vs. Perplexity	36/17	0.013	0.127
ChatGPT vs. Copilot	32/20	0.126	0.505
Copilot vs. Gemini	7/0	0.016	0.127
Copilot vs. Grok	7/0	0.016	0.127
Copilot vs. Perplexity	9/2	0.065	0.327
Gemini vs. Grok	0/0	1.000	1.000
Gemini vs. Perplexity	2/2	1.000	1.000
Grok vs. Perplexity	2/2	1.000	1.000

b denotes cases classified correctly by the first service and incorrectly by the second, and c the reverse. *p* values are derived from exact McNemar tests, and the Holm correction was applied across the ten pairwise comparisons.

**Table 8:** Accuracy of the AI chatbot services according to the side of the impacted tooth.

AI Chatbot Service	Tooth 48 (Right, *n* = 63), Accuracy % (95% CI)	Tooth 38 (Left, *n* = 51), Accuracy % (95% CI)	*p*
ChatGPT	41.3 (30.0–53.6)	41.2 (28.8–54.8)	1.000
Gemini	22.2 (13.7–33.9)	27.5 (17.1–40.9)	0.662
Perplexity	25.4 (16.3–37.3)	23.5 (14.0–36.8)	1.000
Copilot	33.3 (22.9–45.6)	27.5 (17.1–40.9)	0.545
Grok	22.2 (13.7–33.9)	27.5 (17.1–40.9)	0.662

Accuracy values are expressed as percentages with 95% Wilson confidence intervals, relative to the reference standard, stratified by right (tooth 48) and left (tooth 38) mandibular third molars. The two groups are independent, so accuracies were compared with Fisher’s exact test.

## Data Availability

The case-level dataset supporting the findings of this study, comprising the adjudicated reference label for every tooth, the verbatim response of each chatbot service and the analysis code, is available from the corresponding author upon reasonable request. The deidentified image composites cannot be shared publicly because the ethics approval covers their use for the present research only; access to the images may be granted for verification purposes under a data-use agreement following approval by the Biruni University Scientific Research Ethics Committee.
